# An adapted process to develop customized assessments to measure the impact of biology lessons

**DOI:** 10.1093/iob/obag034

**Published:** 2026-07-01

**Authors:** A E Foltz, S Z Hoagland, A N Olson, B N MacNeill, M R Stetzer, M K Smith, B A Couch

**Affiliations:** Department of Physics and Astronomy, University of Maine, Orono, ME 04469, USA; School of Biological Sciences, University of Nebraska, Lincoln, NE 68588, USA; School of Biological Sciences, University of Nebraska, Lincoln, NE 68588, USA; Department of Ecology and Evolutionary Biology, Cornell University, Ithaca, NY 14853, USA; Department of Physics and Astronomy, University of Maine, Orono, ME 04469, USA; Department of Ecology and Evolutionary Biology, Cornell University, Ithaca, NY 14853, USA; School of Biological Sciences, University of Nebraska, Lincoln, NE 68588, USA

## Abstract

When instructors, curriculum developers, or education researchers implement an innovative lesson or develop a shareable lesson plan, they likely want to know if the lesson is effective at facilitating student learning. Published assessments, which have undergone a substantial development and optimization process, are commonly used to evaluate the impact of curricular and pedagogical innovations. However, there are some cases when using an existing concept assessment does not align with the goals of the person administering the assessment. Concept assessments usually cover a broad content domain spanning entire units, courses, or majors. If an instructor, curriculum developer, or researcher intends to assess learning associated with a discrete time period (e.g., a lesson), a concept assessment may be too broad and thus lack alignment validity. Consequently, they may decide to develop their own assessment. They can accomplish this by writing original questions, using question banks, using questions provided in open educational resources, or a combination of these approaches. Alternatively, they may pursue a time- and resource-intensive comprehensive assessment development process, typically involving several steps to accrue validity evidence. There are currently no guidelines for scaling the process for contexts in which existing concept assessments are misaligned and a comprehensive assessment development process is impractical. Here, we describe an adapted assessment development process to generate lesson-customized assessments. We started with five published biology lessons, covering a broad range of biology topics, and created a lesson-customized assessment for each. Our assessment development process consisted of identifying lesson learning objectives, drafting an initial assessment, soliciting expert feedback and student interviews, and conducting several cycles of review and revision. We provide suggestions to help with practicality and make the process more accessible. Though we developed customized assessments for published biology lessons, our approach is broadly applicable to other cases where customized assessments may be beneficial.

## Introduction

Assessments are commonly used by biology instructors and education researchers to gauge students’ conceptual knowledge of a given topic and to identify gaps in understanding. Assessments can be administered before an instructional period (e.g., a lesson, intervention, unit, course) to determine students’ incoming knowledge and again after the instructional period to ascertain what students have learned as a result of instruction (Furrow & Hsu 2019). Concept assessments are a type of assessment that has gained prominence in the biology education field over recent decades (Branchaw *et al.* 2020). Concept assessments typically undergo a development process consisting of several steps that support valid and accurate measurements of conceptual understanding (i.e., the process optimizes the ability of assessments to measure what they intend to measure; Messick 1995a; Adams & Wieman 2011; American Educational Research Association, American Psychological Association, and National Council on Measurement in Education 2011; Reeves & Marbach-Ad 2016).

Concept assessment developers begin the process by using or creating a disciplinary content framework, discerning which topics students find difficult, and soliciting feedback from experts to further define the content domain. The developers then gather feedback from experts to ensure content accuracy and from students to ensure that they interpret questions as the developers intend and provide answers that reflect their conceptual understanding. To support using the assessment on a broad scale (e.g., nationally across undergraduate biology courses), developers solicit data from a large and diverse sample of students.

### Challenges with identifying an appropriate assessment instrument

When an investigator (which can refer to an instructor, curriculum developer, researcher, or other scholar using an assessment instrument to collect data about student learning) wishes to assess the efficacy of a particular lesson, unit, or course, they have multiple assessment options. One option is to administer an existing concept assessment. Concept assessments can be administered as is and can produce scores that are reflective of underlying conceptual understanding, since the developers collected validity evidence to support results interpretation. Typically, published assessments have content domains with a broad conceptual focus, spanning entire units, courses, or majors (Kalas *et al.* 2013; Price *et al.* 2014; Smith *et al.* 2008; Summers *et al.* 2018).

Due to this scope, there can be cases where the use of concept assessments does not align with the goals of the investigator (Bass *et al.* 2016; Olson *et al.* 2026), such as when they intend to collect fine-grained data on student learning associated with a particular instructional period. For example, an instructor may want to interrogate a particular aspect of their course. They may find that students struggle with a specific topic, so they want to implement other teaching practices and measure associated learning to determine whether their new approach benefited students. A curriculum developer may intend to collect data on a lesson or other type of instructional material that they will publish or otherwise share with others. A researcher may wish to assess student learning across a variety of topics, such as when investigating the impact of a specific pedagogical approach across many different courses. Lastly, instructors engaged in the scholarship of teaching and learning (SoTL) or educational action research may find it useful to collect learning data that can inform ongoing instructional decisions.

In each of the aforementioned cases, an existing concept assessment may not suffice due to misalignment between the content of an instructional period and the assessment content. The size, complexity, and diversity of biology as a discipline leads to vast course content options that exceed instructional bandwidth. As a result, instructors develop instruction that reflects instructional needs, instructional constraints, unique interest areas, and innovative content combinations (e.g., lessons that focus on cross-disciplinary topics). As assessment options tend to favor canonical topic areas, assessments with sufficient alignment to the lesson may be unavailable.

### The assessment needs to align with the content of the instructional period

Alignment is a form of validity evidence that has been defined as “the degree to which the scope, content, focus, phrasing, and presentation of an instructional period aligns with the corresponding assessment and intended learning objectives (LOs)” (Olson *et al.* 2026). Alignment validity is distinct from the traditional forms of validity, though it is closely related. For example, both content and alignment validity focus on aligning the assessment to what the developers intend to measure. However, content validity focuses more on the assessment instrument, in terms of its scientific accuracy and the degree to which the assessment mirrors and spans the underlying construct. Alignment validity recognizes that instruction does not always cleanly align with a defined content domain and thus focuses on the alignment between the assessment and the instructional period being assessed. Alignment validity is especially important when an instructor has already developed the content for an instructional period and is considering whether to administer a particular assessment. Importantly, poor alignment may result in student scores that do not accurately reflect their understanding of concepts targeted by the instructional period (Messick 1995b). Thus, using a misaligned assessment can lead to misestimation of learning gains and thus misestimation of instructional period efficacy (Olson *et al.* 2026). Consideration of all forms of validity, including alignment validity, is key in determining if the intended goals of the instructional period are met (i.e., are students learning what we want them to learn from the instructional period?).

Given the potential misalignment between an existing concept assessment and a target instructional period, an investigator may choose to develop their own assessment. The time and resources devoted to developing such an assessment can range from minimal to extensive. They may decide to write their own assessment questions or compile them from other sources (e.g., past course materials, online question banks). This approach can be useful, because it allows the assessment to have alignment validity. However, the assessment will lack other forms of validity, such as the extent to which students interpret questions as intended and whether their answers reflect their underlying conceptual understanding (Messick 1995b). Alternatively, they may pursue a comprehensive assessment development process, which will provide validity evidence to support the interpretation of the assessment results. In this example, this approach also allows for the assessment to have alignment validity because the investigator can tailor the assessment to the content covered in the instructional period. However, this process is not always feasible, as it is time-consuming and requires significant resources (e.g., personnel and funding). Developing an assessment can be a multi-year process, so some situations require an expedited process. Thus, between the options of writing an assessment with little validity evidence or pursuing comprehensive assessment development, the investigator can match the resources available to develop the assessment to their intended goals of the assessment. Developing a customized assessment allows the investigator to collect and optimize validity evidence, including alignment validity, to an appropriate degree without undergoing the years-long process of comprehensive assessment development. We aimed to adapt the existing assessment development process to generate what we refer to as lesson-customized assessments, or assessments developed to be aligned to our given instructional period (i.e., a lesson).

### Developing assessment instruments for OER lessons

We adapted a development process to generate assessments aligned to specific lessons. These published biology lesson plans are a form of open-educational resources (OERs). OERs are free and easily-accessible educational resources, such as textbooks, videos, lab activities, or lesson plans (Ahmed *et al.* 2024). Biology OER lesson plans can be published in peer-reviewed journals (e.g., CourseSource: https://qubeshub.org/community/groups/coursesource; Journal of Microbiology and Biology Education: https://journals.asm.org/journal/jmbe; Teaching Issues and Experiments in Ecology: https://tiee.esa.org/). Though OER lesson plans commonly include questions that can be used during class (e.g., clicker questions) or during exams, they generally do not include a comprehensive set of questions that cover all LOs or lesson topics. Also, it is rare for OER lesson plans to include robust assessment instruments that have undergone validity testing, though there are a few noteworthy exceptions (e.g., Marbach-Ad *et al.* 2007, 2009, 2010; Andrews *et al.* 2012; Price *et al.* 2014). We chose to focus on OER lesson plans because they correspond to discrete instructional periods and typically lack robust associated assessments. Additionally, OER lessons are ideal for lesson-customized assessment development, because they are widely available and represent innovative teaching strategies (Treibergs *et al.* 2025). In practice, OER lesson-customized assessments can be used to measure student conceptual understanding of instructional content. Additionally, the OER lesson and associated assessment can be administered widely across courses and institutions to assess the efficacy of the potentially varied teaching practices utilized while implementing the lesson.

**Fig. 1:**
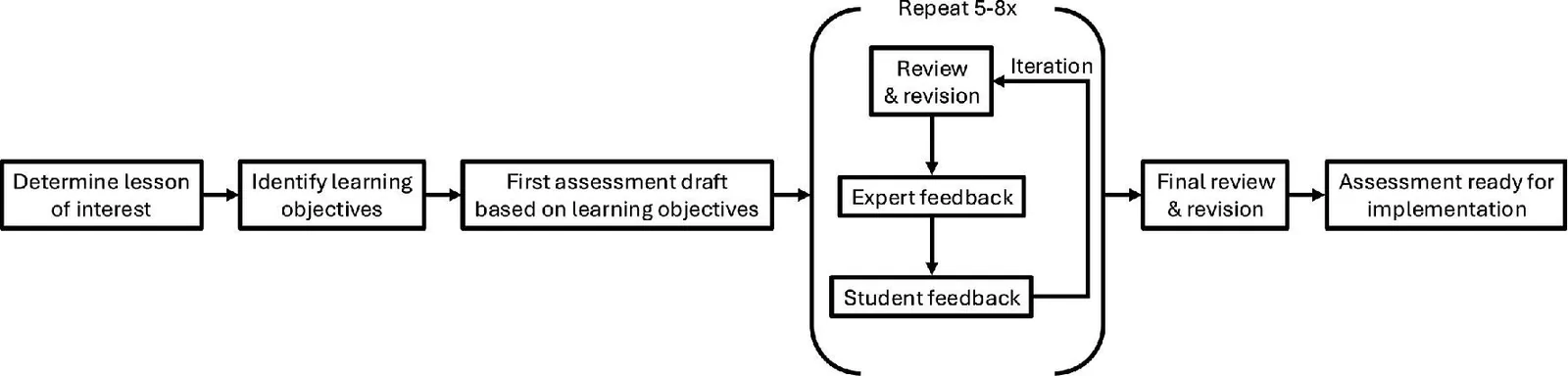
Lesson-customized assessment development process. Note that the review and revision step includes iteration, which helps refine question content and can be calibrated based on an investigator’s access to expert and student feedback.

Though there are resources to aid in developing concept assessments (Adams & Wieman 2011; American Educational Research Association, American Psychological Association, and National Council on Measurement in Education 2011; Bass *et al.* 2016; Tuma & Dolan 2025), we adapted these existing assessment development processes to generate lesson-customized assessments. The resulting assessments are appropriate for capturing student learning in contexts where existing concept assessments are misaligned to the lesson. In contrast to existing assessment development frameworks where developers undergo an extensive process of defining a content domain and then building an assessment based on relevant constructs, we built our assessments based on the lesson curricula so that the assessments would be aligned directly to lesson content. Our lesson curricula consisted of five peer-reviewed biology OER lesson plans. We developed an assessment for each using the following process. An overview of the process is shown in Figure 1 and further details of each step can be found in Table 1.

**Table 1:** Detailed overview of the adapted assessment development process, including suggestions for potential adjustments to the process depending on the needs and resources of the investigator.

**Step of the process**	**Lesson-customized assessment development process**	**Potential adjustments to the process**
Identify learning objectives (LOs)	Examine ● Listed LOs ● Lesson materials (e.g., slides, worksheets, clicker questions) ● Misconceptions addressed by the lesson	May also consider ● Concepts that the investigator has noticed students struggle with ● Content that the investigator chooses to emphasize when teaching the lesson
First assessment draft	Considerations for writing questions ● Question stem: o Use diverse organisms (Heredia *et al.* 2016) o Use a descriptive scenario ● Data/diagram/model: o Can use different formats (e.g., figures, tables, graphs) ● Statements (Adams & Wieman, 2011; Frey *et al.* 2005): o Avoid absolute words and double negatives o Avoid multiple clauses in one statement o Can address multiple LOs across multiple statements o Write more statements than needed in the final assessment	Initial draft questions can also be compiled from ● Existing materials from a lesson (e.g., example exam questions) ● Past course materials ● Online question banks (e.g., https://oercommons.org/) ● Open-source textbooks (e.g., https://openstax.org/)
Review and revision	● Individual reviews and team discussions ● Make note of any outstanding within-team disagreements (to monitor during subsequent *expert feedback* and/or *student feedback* steps) ● Complete this step iteratively as more expert and student feedback is acquired ● Stop revising when feedback saturation is reached	● For feedback on assessment structure, consider contacting learning communities or centers for teaching and learning ● Rather than incorporating expert feedback as a separate step, it can be included as part of the initial review
Expert feedback	● Experts include individuals with proficiency in the lesson topic (e.g., lesson authors, faculty, postdocs, and/or graduate students ● Share current assessment draft with the expert and ask them to answer the following about each assessment question: o Is it clear what the item is asking? o Do you agree that the selected answer is correct? o Are the item and the answer scientifically accurate? o Is this an appropriate question to ask a lower-division biology student? o Is there anything else that the assessment developers should change about the item?	● Experts can also include colleagues, former collaborators, and/or teaching assistants ● Trade assessments with another instructor, curriculum developer, or researcher with expertise on the lesson topic
Student feedback	Student interviews (Adams & Weiman, 2011) ● Individual student with an interviewer ● Students take most recent assessment draft, “attend the lesson,” then re-take most recent assessment draft while engaging in a thought-process think-aloud interview ● Student reads questions aloud and talks through their reasoning as they answer each question	Interview adjustments ● May need to avoid conducting interviews with active students ● If conducting interviews with active students is preferred, it may be necessary to train a research assistant to conduct interviews
	● Interviewer can remind student to think aloud but should mostly just give the student space to think and speak ● Interviewer asks follow-up questions ● Whole-assessment follow-up questions (e.g., if any words were unfamiliar, if all questions were of appropriate difficulty) ● Statement-specific follow-up questions (clarify what information the student used to answer the question, what made them unsure of their answer if they guessed)	Alternatives to interviews ● Pilot the lesson and pre- and post-lesson assessments in a course ● Ask for student feedback (e.g., verbally, through an open-response post-assessment question, via notecards in-class), revise assessment before next academic term ● This can help address the issue that high-performing students tend to participate in interviews ● If you have undergraduate learning assistants or teaching assistants, they can provide a student perspective
Final review and revision	● When feedback saturation is reached, conduct final within-team review and revisions ● Remove extra statements that are not performing as intended	

## A refined process for developing lesson-customized assessments

### Identify lesson of interest

To develop lesson-customized assessments, we first selected five *CourseSource* lessons. The criteria we used when selecting lessons included a strong download history, feasibility to complete in 1–2 class sessions, and relevance to Vision and Change (V&C) core concepts (AAAS 2011). We also selected lessons representative of a broad range of biology topics. We chose articles that focus on the central dogma of molecular biology (Pelletreau *et al.* 2016), population growth models (Trenckmann *et al.* 2017), cellular respiration (Freeman *et al.* 2017), cell structure and function (Sestero *et al.* 2014), and coevolution (Hoskinson *et al.* 2014). We developed the five assessments individually by modifying the phases and steps commonly used in comprehensive assessment development (Knight 2010; Adams & Wieman 2011; American Educational Research Association, American Psychological Association, and National Council on Measurement in Education 2011). The following phases describe the development process for a single lesson-customized assessment.

### Determine what content is included during the instructional period

After identifying the lessons of interest, we determined the specific topics students will engage with and learn during an individual lesson. To do this, we read the article in its entirety, taking notes on the different aspects of the lesson, including the intended audience, in-class activities, and any supplemental materials, which often include lesson slides, worksheets, or clicker questions. We especially focused on defining the LOs of the lesson. Some journals, like *CourseSource*, require authors to articulate LOs, which are listed near the beginning of the article. We also identified other LOs that were prominent in the article. These were concepts that would be taught during the lesson but did not have a previously articulated LO associated with them. For example, how the lesson addresses a misconception might not be explicitly stated as a LO, but students learn about the concept in a way that clarifies common student misconceptions. In the case of the coevolution lesson, the lesson emphasized adaptation as both a biological process, due to natural or artificial selection, and an outcome, such as a change in allele frequency or a change in a measure of fitness. Teaching students about adaptation as a process and an outcome addresses the misconception that a species has the agency to choose to adapt or not adapt. This misconception, however, is not directly stated as an LO in the OER (Hoskinson *et al.* 2014). After identifying all LOs in a lesson, we used them to construct the first draft of each corresponding assessment.

### Draft initial assessment

While constructing the initial draft of the assessment, we used a closed-ended multiple-true-false (MTF) question format (Adams & Wieman 2011). Previous work has shown that MTF helps instructors better diagnose student thinking compared to other closed-ended formats, such as multiple-choice (Hubbard *et al.* 2017; Couch *et al.* 2018; Brassil & Couch 2019). Each question stem presented a unique scenario and included any figures, tables, or graphics students needed to answer the associated statements. Across the stems, we used diverse organisms, as students might have preconceived notions about organisms they are more familiar with or might view the stem with a different lens depending on the organism presented (Heredia *et al.* 2016). We avoided using absolute words, double negatives, and multiple clauses in one statement as they can influence how a student answers the question (Frey *et al.* 2005; Adams & Wieman 2011). When drafting the initial assessment, we wrote more statements than necessary, anticipating that some would be removed during the review and revision phase.

### Review and revise assessment

The assessment went through several iterations with revisions informed by extensive feedback. The initial review process involved members of the research team, who have a range of biology expertise and teaching experience, individually reviewing the assessment and making suggestions for how to improve the question stems and associated statements. The team then discussed the comments as a group and made edits based on the comments and discussion until we reached a consensus. Any uncertainty was noted and would be addressed later in the review and revision phase.

After the first round of revisions, we continued the review and revision phase with expert feedback and student interviews, broadly mirroring established assessment development frameworks (Knight 2010; Adams & Wieman 2011; American Educational Research Association, American Psychological Association, and National Council on Measurement in Education 2011). The feedback received from student interviews and experts helped guide iterative revisions of the question stems and statements. After expert feedback was received or upon completion of a student interview, our research team reviewed and discussed the feedback and decided what changes to make.

We contacted a variety of experts for feedback on the assessments. Each expert had proficiency in the lesson topic and included lesson authors, faculty, postdocs, and graduate students from a variety of institutions. We shared the current assessment draft with the expert and asked them to focus on several questions as they went through each assessment question:

1. Is it clear what the item is asking?
2. Do you agree that the selected answer is correct?
3. Are the item and the answer scientifically accurate?
4. Is this an appropriate question to ask a lower-division biology student?
5. Is there anything else that the assessment developers should change about the item?

Expert feedback often focused on scientific accuracy or the appropriateness for the intended lesson audience. This feedback usually resulted in our team referring back to the original lesson material to ensure that any revisions would maintain alignment between the assessment and the lesson.

The goal of student interviews was to understand the thought processes of students as they worked through the questions, determine if the assessments were accurately capturing student thinking, and identify areas needing revision (Adams & Wieman 2011). Each student was interviewed individually. Students took the most recent version of the assessment at the beginning of the interview. Students were not taught these lessons in previous courses, therefore we simulated the classroom environment by having them watch a recorded lesson video. The video consisted of one of the team members teaching the lesson using the slides provided as supplementary material from the published lesson. During the video, the student interviewees were asked to engage with the lesson as if they were in a classroom setting by pausing periodically to work through activities. For the sake of these interviews, the only modifications made to the lesson were related to in-class student activities as only one student was interviewed at a time. If the original lesson included group collaboration, the recorded lesson included the activity and prompted the student to pause the video to engage with the activity individually.

Once the lesson video was over, students took the assessment again, using a think-aloud interview format. The think-aloud interview required students to read the questions aloud, verbally walk through their reasoning while answering the questions, and state what answer they chose. After students finished the assessment, they were asked follow-up questions to ensure the interviewer understood the student’s thought process (Adams & Wieman 2011). All students were also asked follow-up questions pertaining to the whole assessment, such as if there were any words that were unfamiliar and what questions they found to be the easiest or hardest and why. These questions helped the interviewer learn if questions or statements were at an appropriate difficulty level for the students. If statement-specific follow-ups were needed, students were informed that the interviewer was not asking because they answered incorrectly but to increase the interviewer’s understanding of the students’ thought processes. Statement-specific follow-up questions were asked to clarify why a student answered a certain way, including what information they used to answer the question or what made them unsure about the answer choices if they guessed.

### Final lesson-customized assessments

The final assessments for each of the five topics included six question stems with four statements each, for a total of 24 statements, and was intended for students to take about 10 to 15 minutes to complete. Student interviews helped ascertain the appropriate number of statements to include to address all LOs without much redundancy (Crocker & Algina 1986). Additionally, the interviews aided in determining the time students needed to complete the assessment. The full concept assessment development process can involve collecting feedback from over 50 experts and conducting up to 200 student interviews (e.g., Couch *et al.* 2019). In this study, for each assessment, we received feedback from 4–6 experts, conducted 6–9 student interviews, and had 12–18 rounds of revision (Table 2). We include guidance in later sections about how investigators might optimize this process based on their particular needs and constraints. The length of time devoted to the development process can vary based on the extent of modification to the process, the number of investigators, and the availability of investigators, students, and experts. We stopped collecting feedback when we were no longer receiving comments on previous issues or concerns and felt that we had reached a saturation point. When this happened, we decided the assessments were finalized. The final assessments (Supplementary File 1) are aligned to the relevant lessons and can be used to detect direct pre-post learning gains due to the implementation of the lesson. Individuals who are interested in using any of the assessments can contact the corresponding author to obtain the relevant answer key(s).

**Table 2:** Amount of expert feedback, student interviews, and iterations of each assessment for each *CourseSource* article selected.

Lesson topic	Central dogma	Population growth models	Cellular respiration	Cell structure and function	Coevolution
Number of expert reviews	4	4	5	5	6
Number of student interviews	9	6	7	8	8
Number of versions	13	15	18	12	14

While test administration and data analyses are beyond the scope of this manuscript, we anticipate that many investigators will want to use classical test theory to analyze their student data (Crocker & Algina 1986; Meguellati *et al.* 2024). While the full concept assessment development process would include multiple rounds of pilot testing (including conducting data analyses on responses), we have removed that from our process as it involves collecting data from a large sample of pilot participants. However, if investigators choose to, they can use *item difficulty indices* to determine if their items sufficiently vary in their relative challenge and *item discrimination indices* to identify items where high performers score noticeably lower than expected, which may indicate a question flaw. *Test reliability* (e.g., Cronbach’s alpha, Kuder-Richardson 20) can be used to determine the extent to which item responses co-vary as would be expected for items covering similar content. They can calculate student pre- and post- scores, and then determine raw or normalized learning changes as measures of the lesson effect (Hake 1998; Marx & Cummings 2007; Nissen *et al.* 2018). Investigators with larger samples and more extensive statistical training can use Rasch or Item Response models to better estimate item and person parameters (Bond & Fox 2007; de Ayala 2009).

## Potential adjustments to the lesson-customized assessment development process

The described process can be adjusted for practicality based on the needs of the investigator (see Table 1 for an overview of potential adjustments to each step). When drafting the initial assessment, it is not essential to generate questions from scratch. Resources such as past course materials (e.g., worksheets, exams, quizzes), open-source textbooks, or online question banks can be used to find questions with alignment to the lesson that the investigator can then modify before taking the questions through the rest of the adapted development process.

After the first draft of the assessment is finished, it is crucial to have others help with the initial review and subsequent revisions. Learning communities or centers for teaching and learning can be resources to find individuals to give feedback on assessment structure. A number of individuals, including colleagues, collaborators, and teaching assistants can provide feedback on the assessment content. Additionally, leveraging broad disciplinary listservs can be a helpful way to find potential expert reviewers. Overall, receiving help from individuals with diverse perspectives and expertise can help create a small team for review and revisions, similar to that of a research team. Though our initial review was done by our research team and we requested expert feedback separately, feedback from experts could also be part of the initial review. One way to facilitate the initial review and expert feedback process is to trade assessments with another instructor, curriculum developer, or researcher with expertise on the topic and give each other feedback.

To make the best use of limited expert time and availability, investigators can consider giving each expert only a subset of assessment questions and only asking for expert feedback on high-quality assessment drafts that have undergone some revision. We also stress the importance of filtering expert feedback so that the questions use language and assumptions reasonable for the target student population and to ensure that the assessment is still aligned to the instructional period of interest. We found that feedback from even one or two experts can substantially improve the quality of the assessment items. As experts have deep disciplinary knowledge and different expertise, it is important to incorporate some form of expert review and feedback.

Another key part of the assessment development process is student interviews. When the assessment is administered in a course, students will take the assessment (i.e., a pre-lesson assessment), engage with the lesson, and then take the assessment again (i.e., a post-lesson assessment). The goal of student interviews is to gain a better understanding of students’ thought processes as they work through the assessment. More specifically, interviews can help determine statements that are confusing, vocabulary that is unfamiliar, and generally what students think of the assessment. When considering interviews, investigators will want to check with their local research compliance office to determine what oversight and safeguards may be necessary given their broader goals, procedures, and context. Investigators should also consider that student availability to participate in interviews can vary throughout the academic year (e.g., students may have limited availability during certain times in the semester and not be on campus during breaks). An instructor should avoid conducting interviews with their own students to avoid complications with their authority position. Additionally, instructors will not want to expose their active students to assessment materials if these same students will take the assessment as part of broader data collection. Instead, investigators can find other students with biology backgrounds on par with the target population (e.g., past students, students in another instructor’s class section, current undergraduate teaching assistants). If investigators are asking former students to be interviewed, they can encourage participation by informing them that their feedback will be used to improve biology education at their institution. Investigators can also find interviewees by posting to listservs at their institution or contacting biology-related communities on campus (e.g., first-year programs, student clubs). Lastly, investigators can also train a research assistant to conduct these interviews, which can alleviate time constraints as well as help interviewees feel more comfortable during the interview.

While we encourage investigators to conduct student interviews to elucidate student understanding of the assessment, this can be time-intensive and impractical for some investigators, so it is important to consider other options for collecting feedback. The lesson and pre- and post-assessments could initially be piloted in a course. This would provide an opportunity to receive pre-post scores and early feedback from students and to record the lesson to show students later when refining the assessment. We have noticed that higher performing students are more likely to volunteer for interviews (e.g., volunteer bias), so collecting feedback from an entire class represents an effective way to make sure information is gathered from students across different performance levels. Some options to do this include giving students a notecard and instructing them to give feedback on 1–3 questions that they found confusing and why, or having an open response question immediately after an online assessment, where students are given an additional point for providing specific feedback on an aspect of the assessment.

These alternative feedback methods could be done in addition to student interviews or could replace student interviews if conducting interviews is not possible. When collecting student feedback in a written format, it is important to make sure students are giving feedback on specific aspects of the assessment (e.g., what is the easiest question, what is the hardest question, why were those questions easy/hard). These are similar to the follow-up questions asked during a student interview. The specific questions asked will depend on what the investigator wants to learn about their students' perceptions of the assessment questions. An investigator might also want to ask clarifying questions based on the written student feedback. Regardless of the method of collecting student feedback, it is critical for the investigator to ask specific questions that will increase their understanding of student thinking and perceived difficulty of the assessment questions. By understanding how students are engaging with the questions, the investigator can then revise the assessment to most accurately capture student content knowledge.

## Other uses of lesson-customized assessments

There are other instances beyond single lessons where customized assessments may be necessary to achieve instructional or research goals. If an investigator intends to prioritize instructional content rather than a particular content domain, a customized assessment may align more with the intended goals than a concept assessment. Instructional content might be prioritized if the investigator intends to assess (1) an instructional period with a cross-disciplinary focus that does not fully align with the content domain of a single published concept assessment, (2) a subset of components of a content domain, or (3) an instructional period that incorporates innovative teaching strategies, where precise assessment data can aid in measuring the efficacy of the strategies.

## Challenges of developing lesson-customized assessments

Lesson-customized assessments are helpful tools, but they are not without challenges. Developing an assessment can be both time- and resource-intensive. An assessment draft is reviewed and revised several times, requiring both time and effort from the investigator, rest of the development team (if applicable), and reviewers. Additionally, the student feedback step of the process can be challenging in that it can be difficult to find appropriate students to interview or ask for feedback. The student feedback process can be especially time-intensive if an investigator is conducting a full student interview. All of these challenges can impede the rate at which an assessment can be developed and then administered. Given these constraints, we suggest implementing this adapted assessment development process under circumstances in which assessment data is needed for a particular instructional period. In these cases, developing a lesson-customized assessment may be beneficial for both the students and the investigator, as it creates a well-aligned instrument that has validity evidence to support the conclusions drawn from the assessment.

## Conclusion

Lesson-customized assessments are used to determine whether a lesson is working and to measure student learning at a finer scale than concept assessments. We designed these assessments with OER lessons because they are easily accessible, typically do not include robust assessments, can be implemented across many classes (and thus the assessment can also be used widely), and they often include innovative teaching strategies whose effectiveness has not been verified empirically. Despite our focus on OER lessons, this process is broadly applicable and can also be used to develop assessments for pre-existing or newly developed lessons. Additionally, the adapted assessment development process can be applied in instances where other types of assessments may not be ideal to capture student learning of a given topic. The adapted assessment development process is flexible in that it can be adapted to suit the intended assessment goals of the investigator. This adaptability can be important for advancing the scholarship of teaching and learning, as it allows instructors and researchers to examine the efficacy of innovative curricula. Once the customized assessment is administered, it can help the investigator determine specific areas where students have strengths or weaknesses (e.g., misconceptions, conceptual difficulties). Together, lessons and lesson-customized assessments work to increase and gauge student learning and to help instructors and researchers determine the efficacy of a lesson or innovative teaching practice.

## Supplementary Material

obag034_Supplemental_File
