# Supplementary material for: An adapted process to develop customized assessments to measure the impact of biology lessons
Source: Integr Org Biol. 2026 Jul 1;8(1):obag034. doi: 10.1093/iob/obag034 (PMC13383078; doi:10.1093/iob/obag034)
Supplement: obag034_Supplemental_File [file obag034_supplemental_file.docx]

Central dogma assessment aligned to Pelletreau *et al*. (2016).

Indicate whether each statement is true or false.

1. A genetically inherited disease, Barth syndrome, is characterized by an enlarged heart, muscle weakness, low white-blood cell count, and other symptoms. A man has Barth syndrome, but his brother does not. When comparing the brothers’ DNA sequences, scientists find several differences in a gene.
   1. T/F A DNA difference that causes Barth syndrome would be considered a

mutation.

- 1. T/F A DNA difference that does not cause any type of disease would be

considered a mutation.

- 1. T/F It is likely that all of the DNA differences in the affected gene sequence

contribute to the disease.

- 1. T/F The impact of each DNA difference is known from the information

given.

1. After cell division, a single nucleotide difference is introduced in a gene within a human’s DNA sequence. If this DNA difference:
   1. T/F Is in the middle of an intron, it will likely alter the amino acid

sequence of the polypeptide.

- 1. T/F Is in an exon, it could produce a polypeptide with an unaltered amino

acid sequence.

- 1. T/F Leads to substitution of an amino acid with similar chemical

properties, the resulting protein could still have normal activity.

- 1. T/F Leads to substitution of an amino acid with different chemical

properties at a key site, the resulting protein will likely have abnormal activity.

1. A change in the DNA sequence of the promoter region for a gene could affect:
   1. T/F The frequency of transcription initiation of that gene.
   2. T/F The amount of mRNA produced from that gene.
   3. T/F The amount of corresponding protein made.
   4. T/F The amino acid sequence of the corresponding protein.
2. A single nucleotide change occurs in the middle of a gene. This change results in a TAG sequence in the coding strand, which is the DNA nucleotide triplet that encodes the UAG stop codon.
   1. T/F When DNA is synthesized from this gene, DNA polymerase will read

one base at a time.

- 1. T/F DNA polymerase will stop when it encounters this new triplet

sequence.

- 1. T/F DNA replication will be affected by this change.
  2. T/F This nucleotide change could lead to a different expressed physical

trait.

1. When RNA polymerase carries out transcription of a gene:
   1. T/F It reads the DNA template strand one base at a time.
   2. T/F It will stop transcription when it encounters a triplet sequence that

encodes a stop codon.

- 1. T/F It synthesizes a new strand of DNA.
  2. T/F Translation has already occurred.

1. When a ribosome carries out translation:
   1. T/F It will stop translation when it encounters a triplet sequence that

encodes a stop codon.

- 1. T/F It moves along the mRNA strand in the 3’ to 5’ direction.
  2. T/F It synthesizes a polypeptide made up of amino acids.
  3. T/F The ribosome’s small and large subunits separate from each other

after translation ends.

Population growth models assessment aligned to Trenckmann *et al*. (2017).

Indicate whether each statement is true or false.

1. A population of rainbow trout lives in a freshwater stream, supplied by snowmelt from nearby mountains. One winter, the area experiences a drought with record low snowfall. In the following spring, the stream is half as wide as previous years, but the number of trout remains the same. Compared to winter:
   1. T/F The abundance of rainbow trout is higher in the spring.
   2. T/F The density of rainbow trout is lower in the spring.
   3. T/F The rainbow trout population growth rate is lower in the spring.
   4. T/F The stream likely has a lower carrying capacity for the rainbow trout

population in the spring.

1. Use the graph below to answer this question.


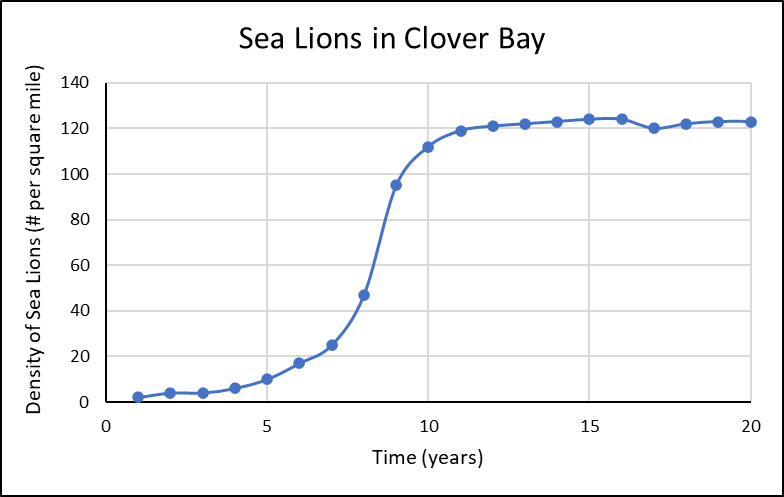


- 1. T/F For the Clover Bay sea lion population, the growth rate is

continuously increasing.

- 1. T/F The Clover Bay sea lion population follows an exponential growth

pattern throughout the entirety of years 1-20.

- 1. T/F The Clover Bay sea lion population has a carrying capacity of about

125 individuals.

- 1. T/F The growth rate is highest around year 8.

1. Researchers observed a small population of koala bears over 10 months. Throughout the observation period, there were zero koala deaths, and one koala was born each month. The koalas had unlimited resources.
   1. T/F A linear growth model would best illustrate the koala population

changes over the 10-month study.

- 1. T/F The koala population growth rate is increasing over the 10-month

study.

- 1. T/F Researchers likely prioritized using density rather than abundance to

estimate the population size due to the small number of individuals in the population.

- 1. T/F The growth rate indicates the koala population is approaching its

carrying capacity.

1. The population of field mice in Elm River State Park is observed for 6 months while it is at carrying capacity. After those 6 months, a pair of red-tailed hawks migrate into the park. Red tailed hawks were not present in the park before, and they predate only on field mice.
   1. T/F During the 6-month observation period, the population size of field

mice most likely decreases.

- 1. T/F During the 6-month observation period, the field mice population

growth rate most likely decreases.

- 1. T/F Following the introduction of the red-tailed hawks, though the field

mice population size decreases, the field mice population growth rate most likely stays the same.

- 1. T/F The introduction of the red-tailed hawks into the park is an example

of a density-dependent regulating mechanism.

1. Four populations of pill bugs were placed in different containers, as shown below.


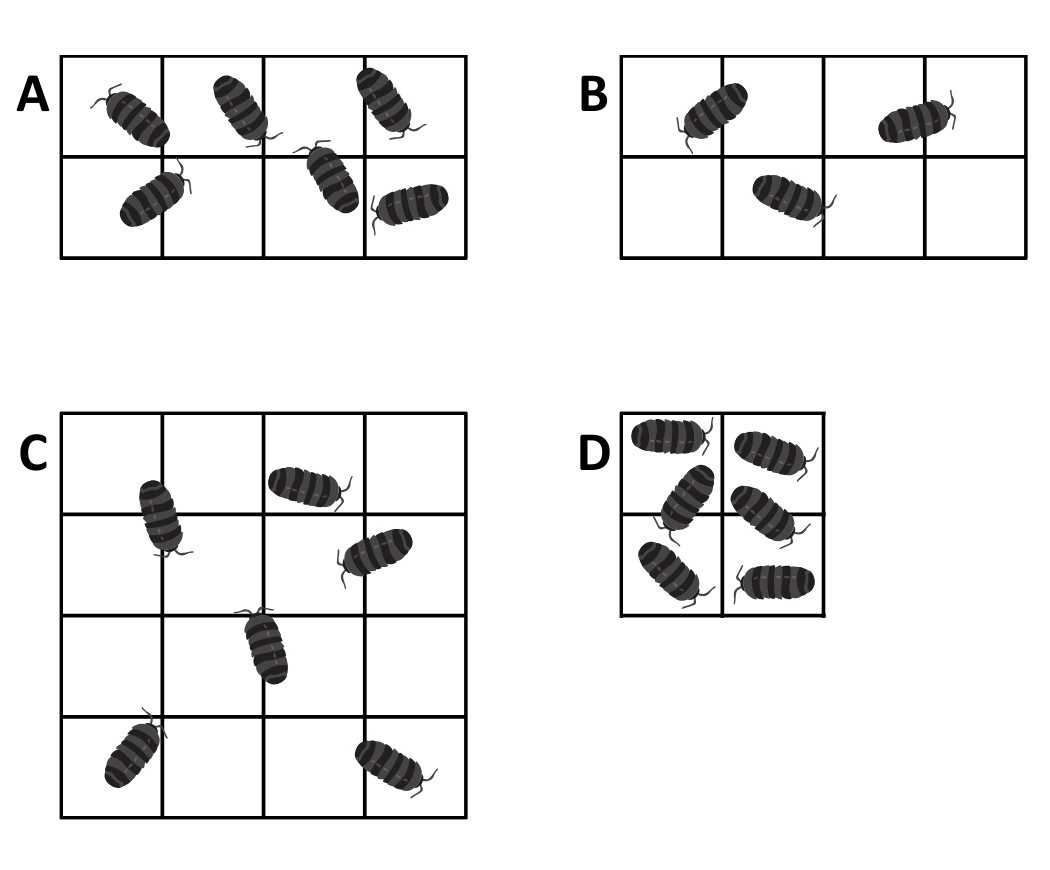


- 1. T/F Population A has the same density as Population C.
  2. T/F Population D has the same abundance as Population A.
  3. T/F Population C has the same density as Population B.
  4. T/F Population D has a greater population size than Population C.

1. The populations of raccoons in Pioneers Park and Wilderness Park are being studied. The population growth pattern of both raccoon populations is best modeled by the equation below. Use the table and equation below to answer this question.


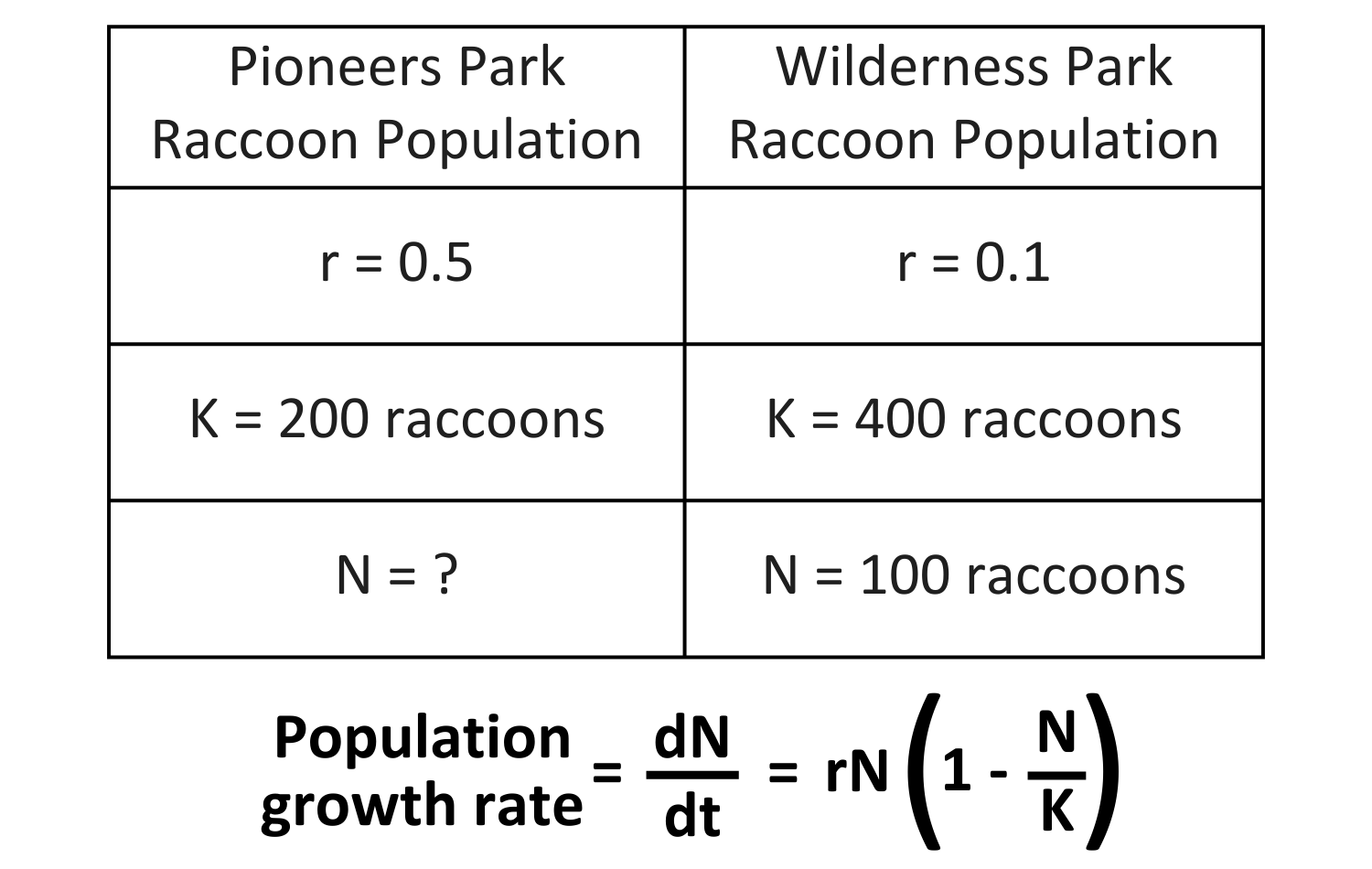


- 1. T/F In Pioneers Park, a population size of 50 raccoons would have a

growth rate of 25 raccoons per year.

- 1. T/F In Wilderness Park, the raccoon population growth rate is less than 10

raccoons per year.

- 1. T/F The population of raccoons in Wilderness Park follows a logistic

growth pattern.

- 1. T/F The difference between raccoon birth rates and raccoon death rates is

greater in Wilderness Park than Pioneers Park.

Cellular respiration assessment aligned to Freeman *et al*. (2017).

Indicate whether each statement is true or false.


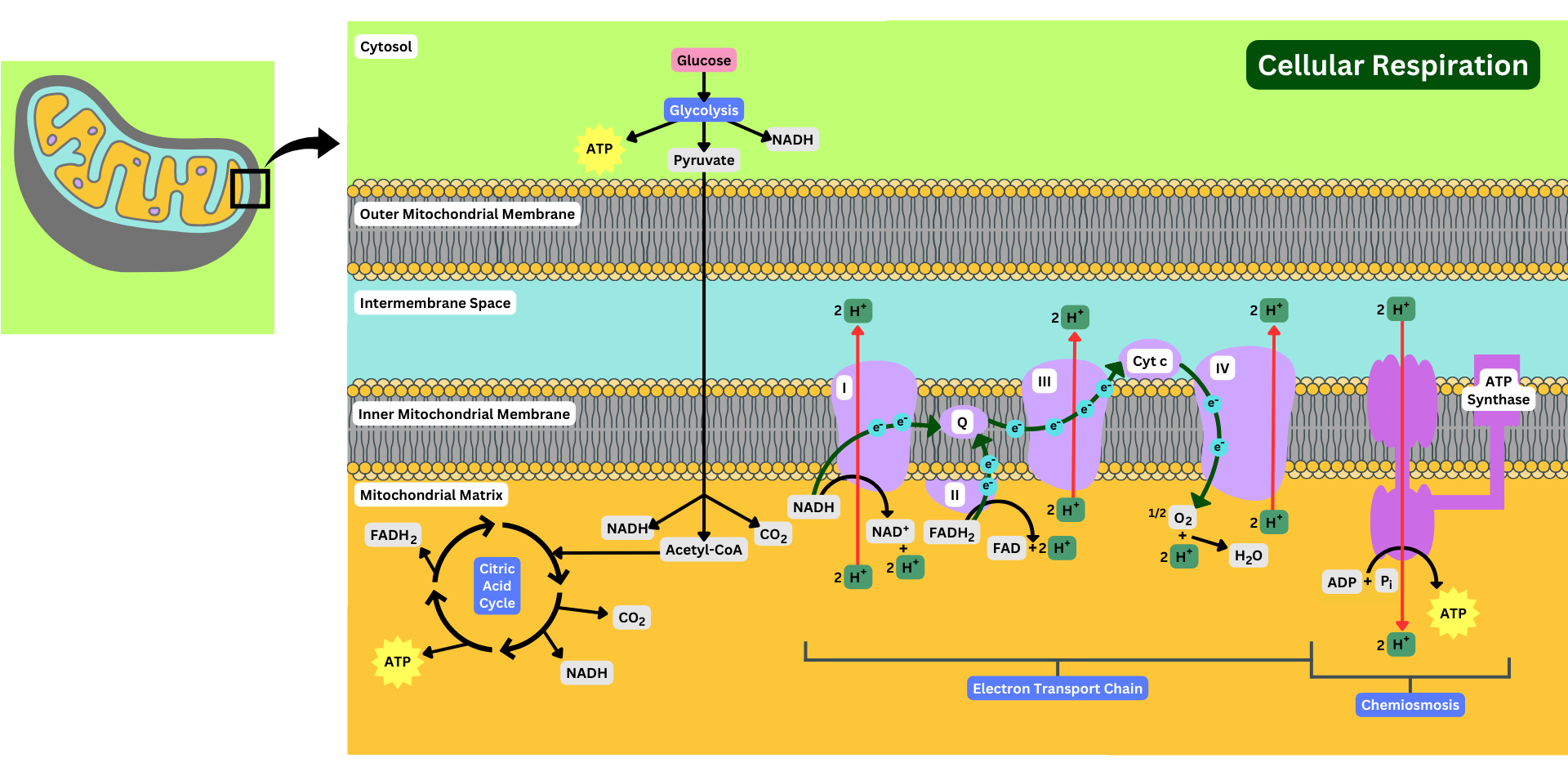


1. ChemX manufactures a wide range of chemicals including wood preservatives and dyes. ChemX often uses hazardous chemicals, including DNP, in the manufacturing process. DNP impacts cellular respiration. A concerned ChemX worker, Dave, arrives in the emergency room and informs the medical staff he accidentally ingested DNP. Dave likely:
   1. T/F Has decreased oxygen levels in his blood.
   2. T/F Has increased glucose oxidation rates.
   3. T/F Produces less ATP than he did before ingesting DNP.
   4. T/F Has increased activity of ATP synthase.
2. A new synthetic drug, A-mell, has been circulating in the body builder community. A-mell is known for quick and dangerous weight loss because it mimics the cellular mechanism of DNP.
   1. T/F A-mell decreases the pH difference between the intermembrane space

and mitochondrial matrix.

- 1. T/F A-mell increases the hydrogen ion concentration of the

intermembrane space.

- 1. T/F A-mell increases the proton gradient between the intermembrane

space and mitochondrial matrix.

- 1. T/F A-mell mainly functions by binding to protons within the

mitochondrial matrix.

1. The table below illustrates the relative levels of different molecules present in a cell.


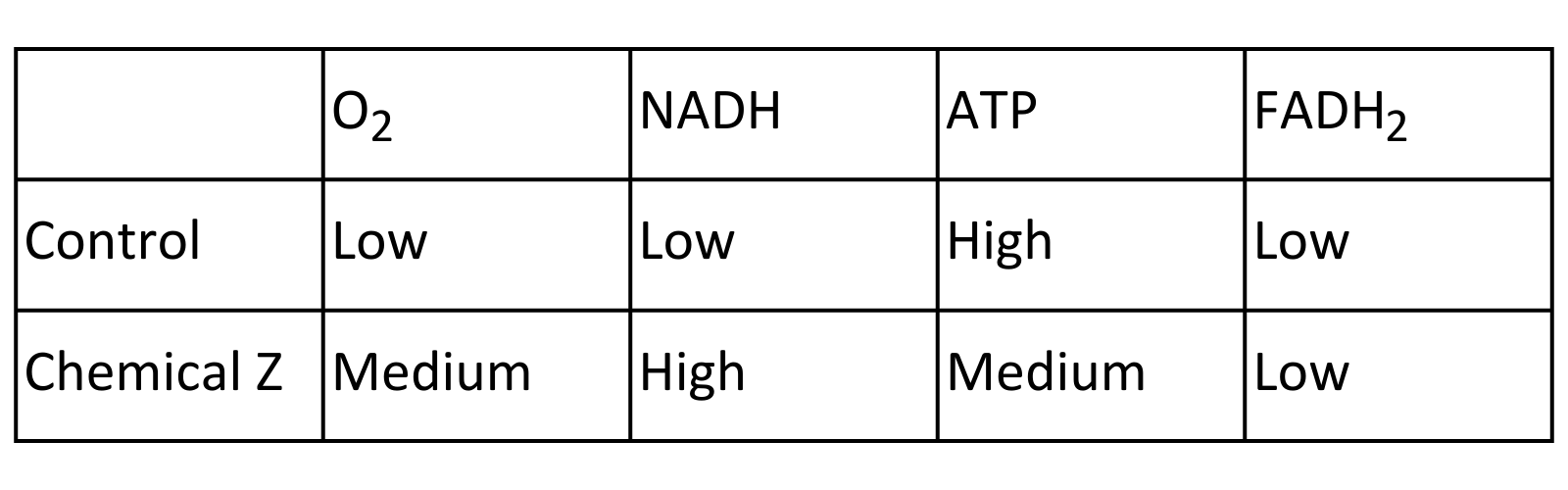


Compared to the control:

- 1. T/F Chemical Z likely results in decreased activity of the electron

transport chain.

- 1. T/F Chemical Z likely blocks a step in the Citric Acid Cycle.
  2. T/F Chemical Z likely disrupts the ability of Complex I to catalyze the

NADH to NAD^+^ reaction.

- 1. T/F Chemical Z likely results in a lower cellular acetyl-CoA concentration.

1. Many reactions are involved in cellular respiration. Answer the following questions about components of those reactions.
   1. T/F During cellular respiration, oxygen is directly converted into ATP.
   2. T/F During cellular respiration, acetyl-CoA is an input in reactions that

lead to the formation of FADH_2_.

- 1. T/F During cellular respiration, NADH is an input in reactions that lead to

the formation of CO_2_.

- 1. T/F ATP functions as a source of energy for a variety of cellular processes.

1. The table on the left shows the mean glucose oxidation rates in myotubule cell cultures treated with and without Chemical C. The graph on the right shows ATP concentrations in myotubule cell cultures treated with and without Chemical C.


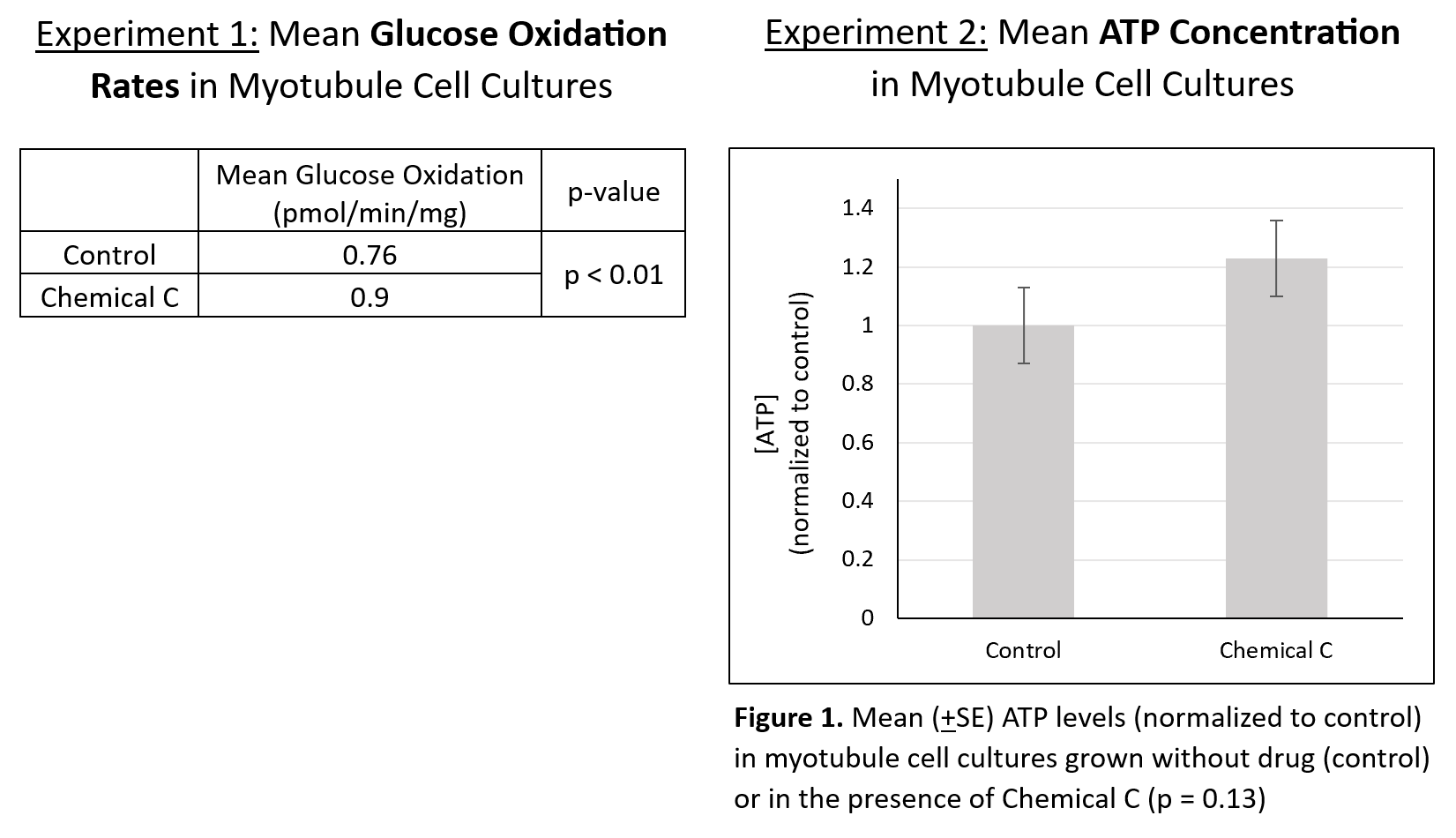


- 1. T/F Chemical C significantly increases the production of ATP compared to

the control group.

- 1. T/F In a control myotubule cell culture, ATP synthase requires energy to

produce ATP.

- 1. T/F Chemiosmosis is likely higher in myotubule cell cultures treated with

Chemical C than in control myotubule cell cultures.

- 1. T/F Glycolysis is likely higher in myotubule cell cultures treated with

Chemical C than in control myotubule cell cultures.

1. A group of mice are temporarily monitored in an air-tight chamber. The solid lines in the graphs below show the changes in the relative concentrations of gases in the chamber under normal conditions.


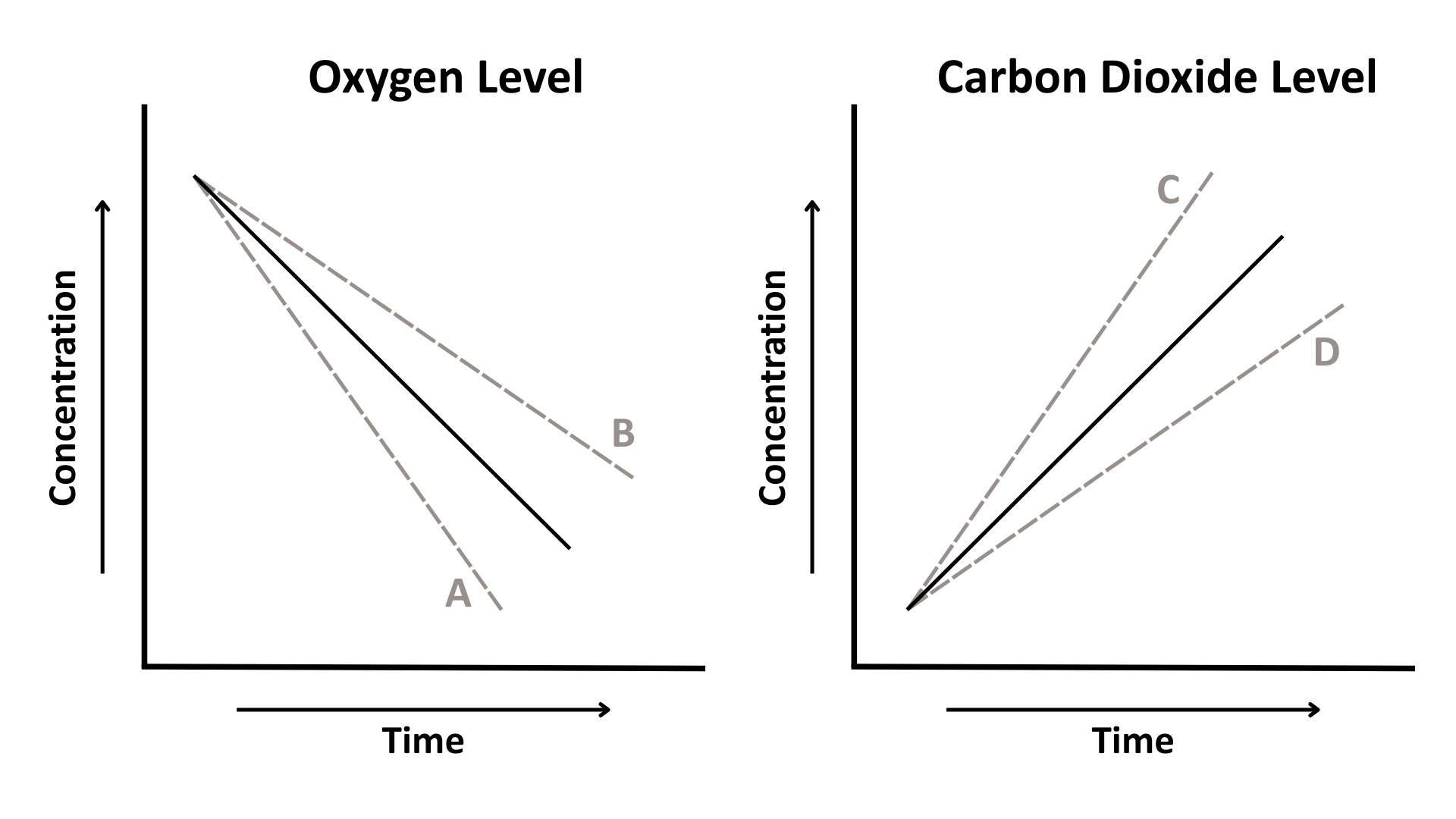


Scientists then examine how substances that target components of cellular respiration impact the mice in the air-tight chamber. They conduct multiple experiments exposing mice to substances of known and unknown function (such as DNP, which causes severe and unsafe weight loss by releasing energy through heat).

- 1. T/F If the mice are exposed to DNP, the slope of the “Carbon Dioxide

Level” graph would be more similar to “C” than “D”.

- 1. T/F If the mice are exposed to DNP, the slope of the “Oxygen Level” graph

would be more similar to “B” than “A”.

- 1. T/F If the mice are exposed to a toxin that prevents the electron transport

chain from functioning, the rate of oxygen consumption would be

higher.

- 1. T/F If exposure to a substance results in a slope of the "Oxygen Level"

graph similar to "A", the slope of the "Carbon Dioxide Level" graph would likely be similar to "D".

Cell structure and function assessment aligned to Sestero *et al*. (2014).

Indicate whether each statement is true or false.

1. Tomato plant cells have different functions based on where they are located in the plant. Tips of tomato plant stems contain cells that require a lot of energy because they are actively dividing. This cell division contributes to the growth in plant height. In contrast, tomato plant leaves contain cells that function to capture energy from the sun. This energy can be converted and used by other cells of the tomato plant, such as cells of tomato plant stems to carry out cell division Based on these different functions, we would expect:
   1. T/F A cell of a tomato plant stem tip likely contains more mitochondria

than a cell of a tomato plant leaf.

- 1. T/F A cell of a tomato plant stem tip likely contains roughly the same

number of lysosomes as a cell of a tomato plant leaf.

- 1. T/F A cell of a tomato plant stem tip likely contains roughly the same

number of chloroplasts as a cell of a tomato plant leaf.

- 1. T/F A cell of a tomato plant stem tip can be distinguished from a cell of a

tomato plant leaf under a microscope.

1. Students used an online graphic design tool to create diagrams of cells with different functions. Use the students’ cell diagrams below to answer this question.
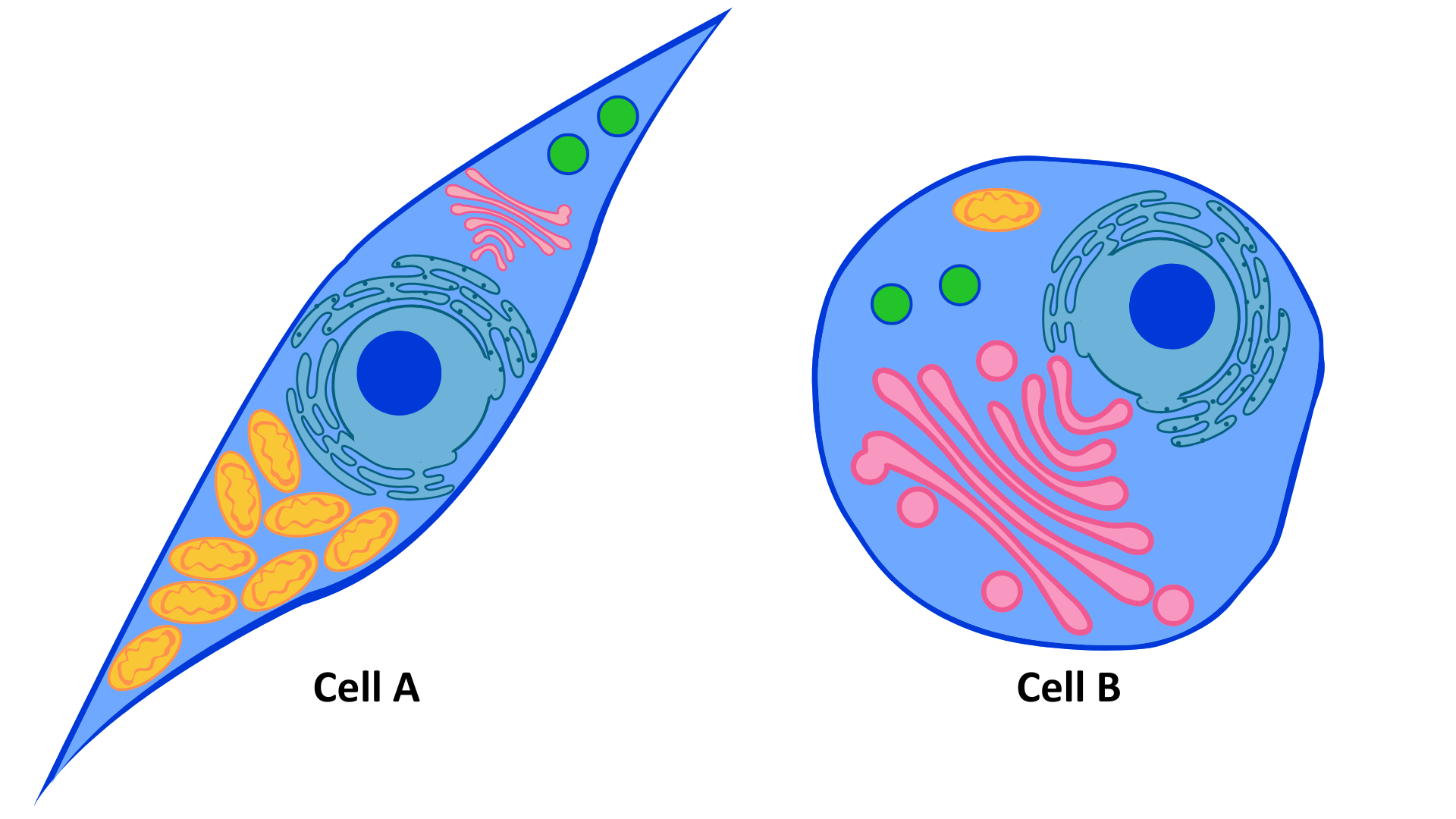

   1. T/F Cell B could be a cell that releases proteins outside of the cell.
   2. T/F Cell B has a cell wall.
   3. T/F Cell A produces a greater amount of ATP than Cell B.
   4. T/F Cell A and Cell B could be cells from the same organism.
2. Use the student drawing of a plant cell below to answer this question.


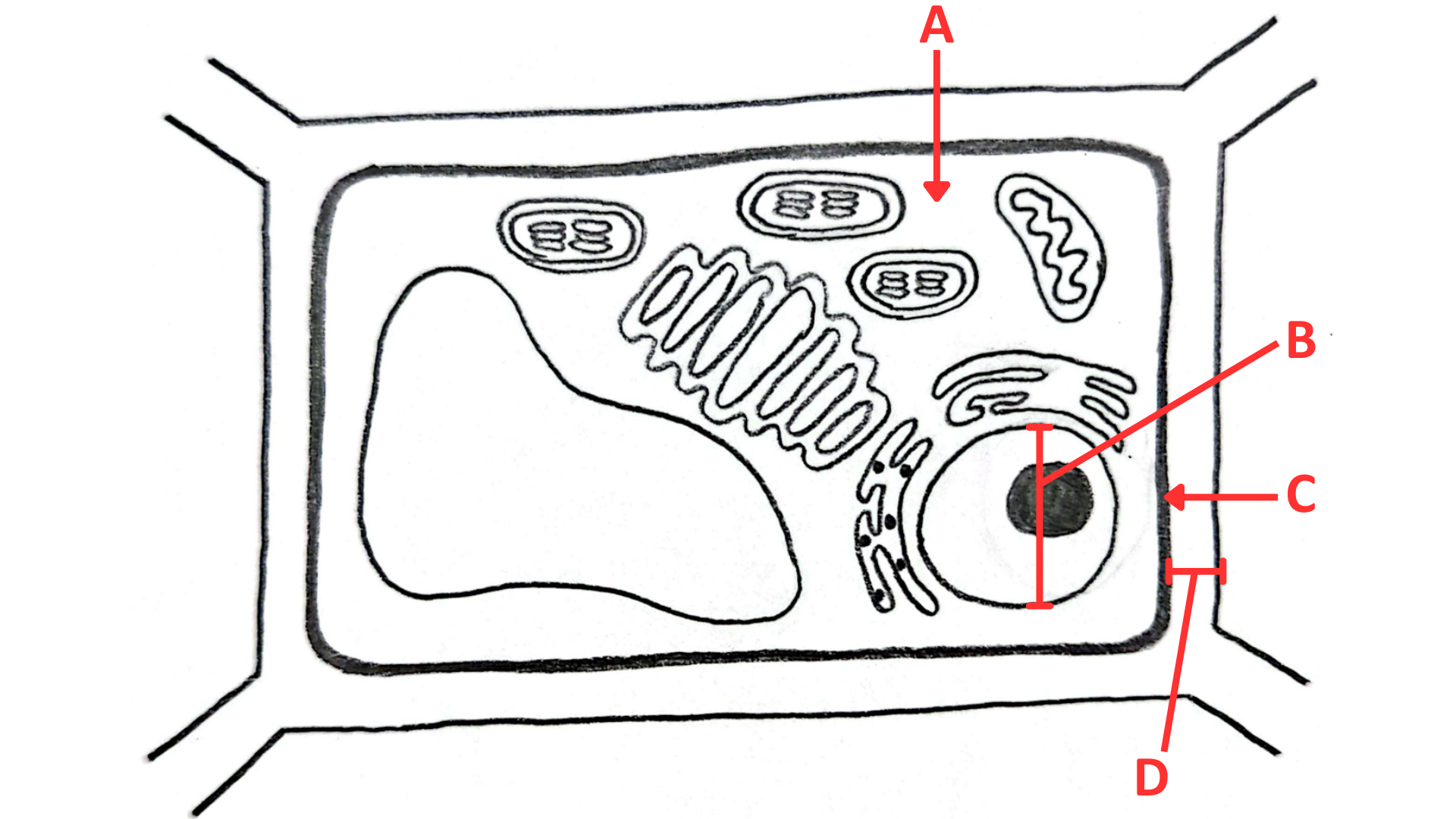


- 1. T/F Structure A is found in plant cells, but not animal cells.
  2. T/F Structure B is found in plant cells, but not animal cells.
  3. T/F Structure C is found in plant cells, but not animal cells.
  4. T/F Structure D is found in plant cells, but not animal cells.

1. The biology department is labeling and digitizing their collection of micrographs depicting various cells. A professor is trying to decipher printed micrographs of two different cells. The micrographs are over 50 years old and have been kept in an old file cabinet, so they are not the best quality. From what the professor can determine:

Cell 1 contains many organelles including but not limited to a large central vacuole, Golgi apparatus, and nucleus.

Cell 2 contains many organelles including but not limited to a cytoskeleton, flagella, and a nucleus.

Based on what the professor knows so far:

- 1. T/F Cell 1 most likely contains centrioles.
  2. T/F Cell 2 is most likely more efficient at movement than Cell 1.
  3. T/F Cell 2 could be an animal cell.
  4. T/F The Golgi apparatus in Cell 1 functions to synthesize proteins.

1. Hormones regulate body functions in humans throughout life. Growth Hormone is a protein hormone produced by the pituitary gland that helps regulate growth in children. Testosterone is a lipid hormone produced by the gonadal glands that helps regulate the production of reproductive tissues and increases blood cell formation. Based on these differences and your knowledge about organelles, you can infer:
   1. T/F A gonadal gland cell likely has a larger smooth endoplasmic reticulum

than a pituitary gland cell.

- 1. T/F A gonadal gland cell likely has more ribosomes than a pituitary gland

cell.

- 1. T/F Growth Hormone is transported through the cytoplasm by vesicles.
  2. T/F The rough endoplasmic reticulum is likely larger in a pituitary gland

cell than in a gonadal gland cell.


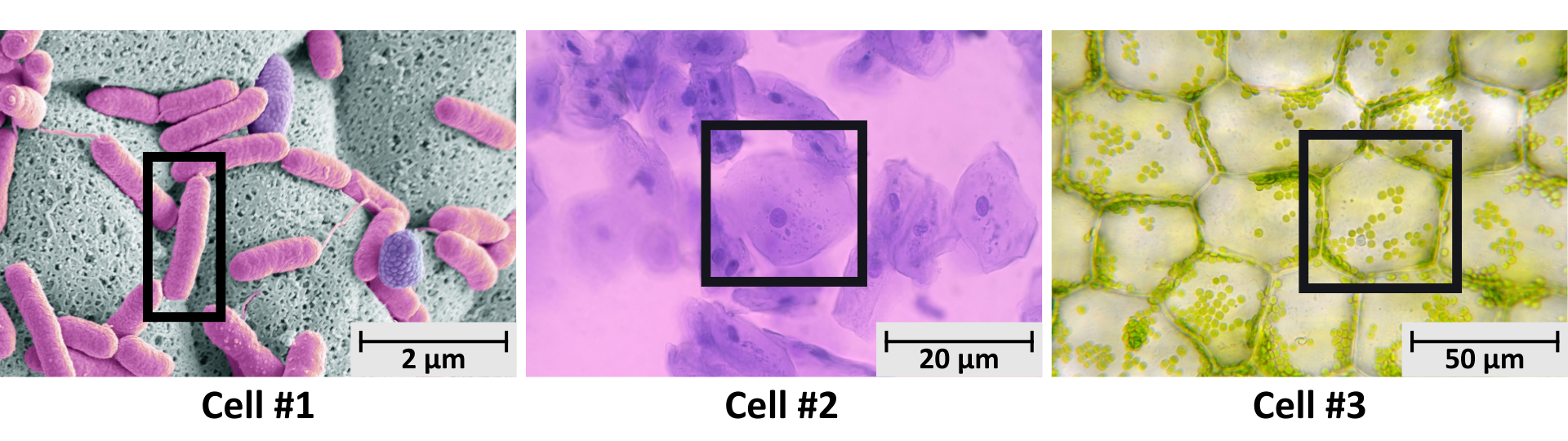


1. Use the images and scale bars below to answer this question.
   1. T/F Cell #1 likely has a greater surface area than Cell #3.
   2. T/F Cell #2 is likely more efficient at holding in water than Cell #3.
   3. T/F Cell #1 contains a nucleus.
   4. T/F Cell #3 is likely a eukaryotic cell.

Coevolution assessment aligned to Hoskinson *et al*. (2014).

Indicate whether each statement is true or false.

1. In adulthood, female spongy moths do not fly and are white in color with or without brown speckles. Spongy moths blend in with the bark of trees where they mate and reproduce. Spongy moths are eaten by birds such as starlings and robins, as well as other predators. Starlings have specialized receptors in their eyes that allow them to distinguish fine details, such as prey blending into the environment. Both moth color and the number of receptors in starling eyes are traits that are passed on from parents to offspring. Answer the following based on this information and the graphs below.
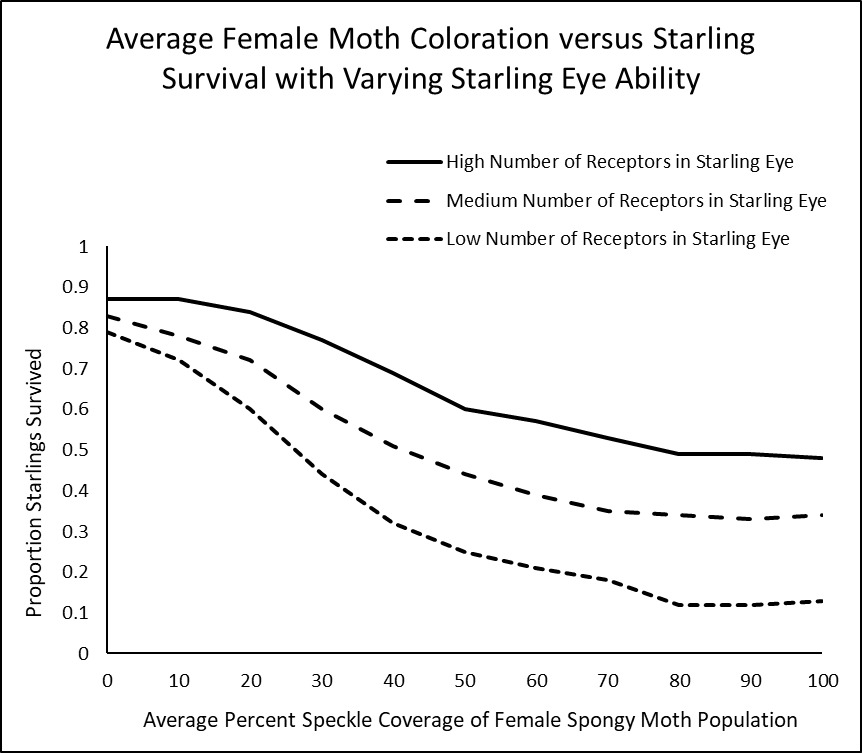


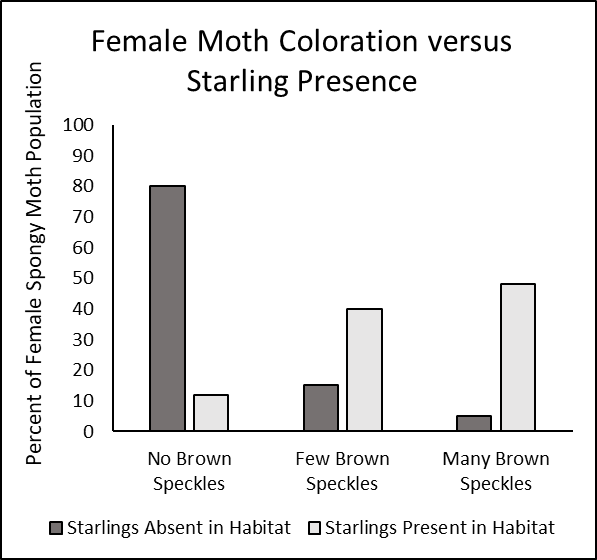


- 1. T/F The evidence suggests coevolution could be occurring between

starlings and spongy moths.

- 1. T/F When starlings are present, female spongy moths with no brown

speckles likely have lower survival than female spongy moths with few brown speckles.

- 1. T/F If starlings are permanently removed from the habitat, the average

percent speckle coverage of female spongy moths will likely decrease over time.

- 1. T/F Spongy moths choose to develop brown speckles when starlings are

present in a habitat.

1. Blue-tailed day geckos are found on the island of Mauritius (Africa). Axolotls are a type of salamander found in Lake Xochimilco in Mexico (North America). Blue-tailed day geckos and axolotls both have a rare survival technique where they can regrow appendages such as limbs or their tail. Mascarene ridged frogs are also found on the island of Mauritius but do not have the survival technique of regrowing appendages.
   1. T/F Blue-tailed day geckos and axolotls likely coevolved the ability to

regrow appendages.

- 1. T/F Axolotls chose to develop the ability to regrow their lost appendages.
  2. T/F If an individual Mascarene ridged frog loses an appendage, it would

adapt by developing the ability to regrow its appendage.

- 1. T/F If blue-tailed day geckos and Mascarene ridged frogs have reciprocal

effects on traits that impact each other’s fitness, coevolution could be

occurring.

1. During a year with average precipitation, the grass in North Creek Prairie is a lush green year round. The grasshoppers in the prairie are typically green but some are yellow-brown. Grasshopper color is passed on from parent to offspring. Green grasshoppers blend in with the lush, green grass of the prairie to avoid predation. Last summer, North Creek Prairie experienced an especially dry summer, causing the grass to turn a yellow-brown and produce fewer seeds. Ecologists studying the effects of the dry summer on the population of grasshoppers in North Creek Prairie might conclude:
   1. T/F During the dry season, an individual grasshopper adapts to its

environment and turns yellow-brown to match the grass.

- 1. T/F Based on the information presented, precipitation is considered a

selective agent that affects the fitness of grass in North Creek Prairie.

- 1. T/F The change of both the grasshoppers and tall grass to a yellow-brown

color provides evidence that a coevolutionary relationship could be

occurring.

- 1. T/F During the dry season, yellow-brown grasshoppers likely have higher

fitness than green grasshoppers.

1. A graduate student is presenting findings from their research project. Their project investigated if coevolution is occurring between a mouse species and an insect species (*Polyplax serrata*) that can live in the fur of the mice. In one of their experiments, the student examined whether exposure to *P. serrata* impacts a population of mice as they progress through multiple reproductive cycles over the course of their lifetimes.

The student is preparing a graph to appropriately present the findings from the data in the table below.

- 1. T/F Average number of offspring per female mouse is an independent
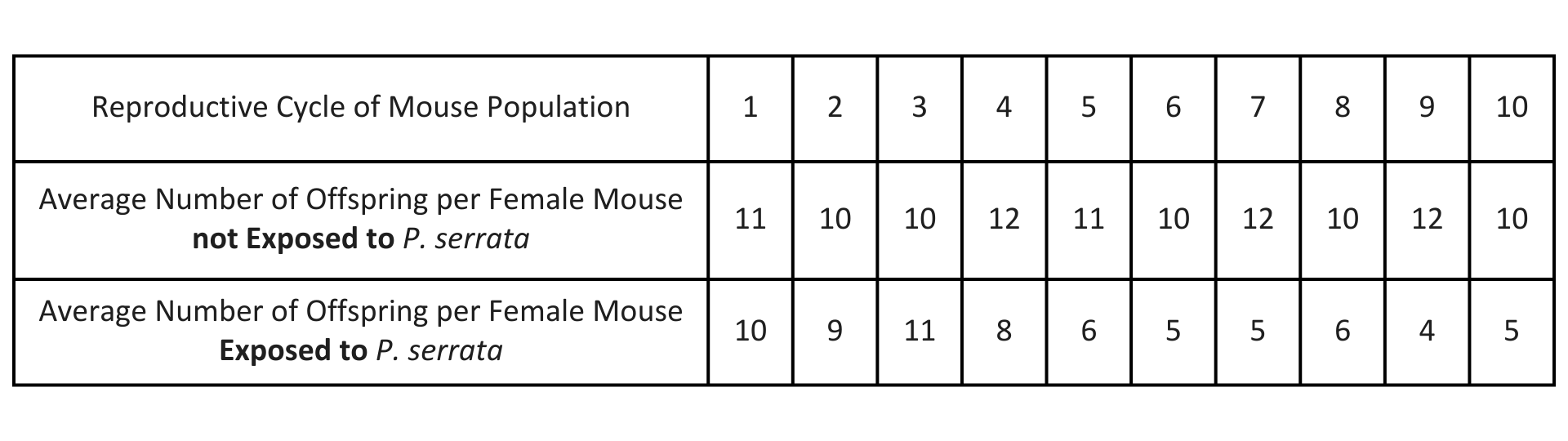


variable.

- 1. T/F The dependent variable should go on the x-axis.
  2. T/F A graph of the data would indicate that mice exposed to *P. serrata*

have a decreasing average number of offspring per female mouse

across reproductive cycles.

- 1. T/F A graph of the data would indicate that mice not exposed to *P. serrata*

have a relatively stable average number of offspring per female mouse across reproductive cycles.


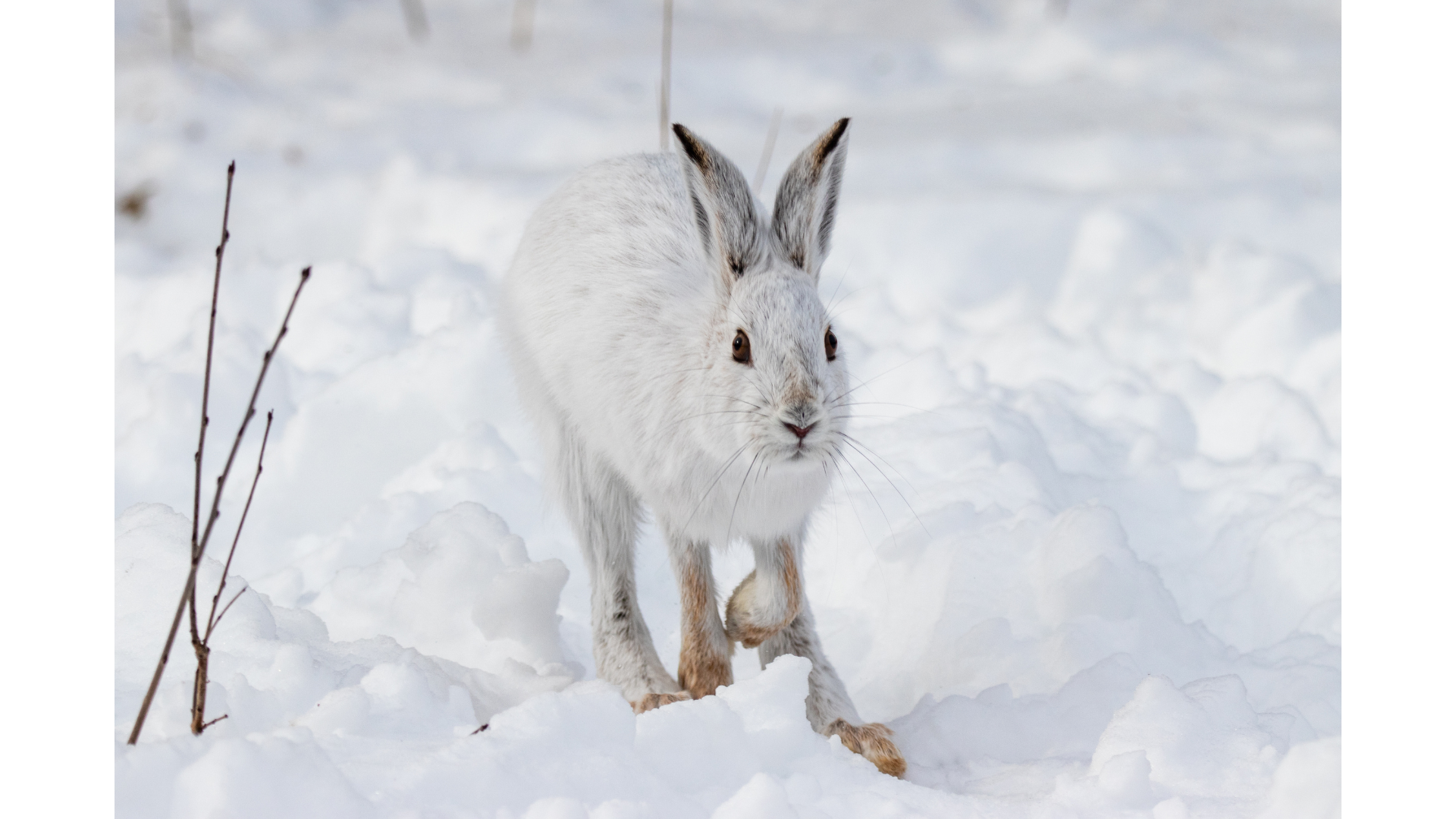


Many species of hares are found throughout the world. Different hare species evolve due to interactions with their environments and other species. Answer the following regarding arctic and snowshoe hares.

- 1. T/F Coevolution can occur between arctic hares in Norway and arctic

hares in Canada.

- 1. T/F Coevolution can occur between an individual snowshoe hare and an

individual arctic hare in the same location.

- 1. T/F If two populations of different hare species are interacting in their

environment, they are coevolving.

- 1. T/F An arctic hare with higher fitness has more surviving offspring than

another arctic hare with lower fitness in the same population.

1. Prairie dogs and other rodents are highly vulnerable to sylvatic plague, caused by the bacterium *Yersinia pestis* found in fleas. When plague-infected fleas feed on a prairie dog, the prairie dog can contract plague and become sick. Without effective control, plague can kill off an entire prairie dog population. Researchers are studying the effects of a current plague outbreak on the prairie dog population in Badlands National Park. One researcher hypothesizes that this outbreak of plague affects prairie dogs more than it affects other rodent species, such as chipmunks. The researcher is considering potential variables involved and the experimental design used to test their hypothesis.
   1. T/F The researcher should compare the plague outbreak in the prairie dog

population in Badlands National Park to the plague outbreak in the prairie dog population in Wind Cave National Park.

- 1. T/F The researcher should compare the plague outbreak on the prairie

dog population in Badlands National Park to the plague outbreak in the chipmunk population in Badlands National Park.

- 1. T/F In Badlands National Park, the vulnerability of rodents to *Yersinia*

*pestis* infection likely affects the fitness of fleas.

- 1. T/F If prairie dogs and fleas are coevolving, then prairie dogs and *Yersinia*

*pestis* are also coevolving.
